# Stylistic features of case reports as a genre of medical discourse

**DOI:** 10.1186/s13256-017-1247-x

**Published:** 2017-03-13

**Authors:** Yuliia Lysanets, Halyna Morokhovets, Olena Bieliaieva

**Affiliations:** 1 Department of Foreign Languages with Latin Language and Medical Terminology of Ukrainian Medical Stomatological Academy, Poltava, Ukraine; 2 Research Department of Ukrainian Medical Stomatological Academy, 23 Shevchenko Str., 36011 Poltava, Ukraine

**Keywords:** Medical discourse, Medical case report, Genre, Style, Lexical and grammatical features

## Abstract

**Background:**

The present paper discusses the lexical and grammatical peculiarities of English language medical case reports, taking into account their communicative purposes and intentions.

**Methods:**

The objective of the research is to clarify the principal mechanisms of producing an effective English language medical case report and thus to provide recommendations and guidelines for medical professionals who will deal with this genre. The analysis of medical case reports will largely focus on the most significant linguistic peculiarities, such as the use of active and passive voice, the choice of particular verb tenses, and pronouns. The selected medical case reports will be considered using methods of lexico-grammatical analysis, quantitative examination, and contextual, structural, narrative, and stylistic analyses.

**Results:**

The research revealed a range of important stylistic features of medical case reports which markedly distinguish them from other genres of medical scientific writing: educational and instructive intentions, conciseness and brevity, direct and personal tone, and material presented in a narrative style. The present research has shown that the communicative strategies of the analyzed discourse, mentioned immediately above, are effectively implemented by means of specific lexical units and grammatical structures: the dominance of active voice sentences, past simple tense, personal pronouns, and modal verbs. The research has also detected the occasional use of the present perfect, present simple, and future simple tenses and passive voice which also serve particular communicative purposes of medical case reports.

**Conclusions:**

Medical case reports possess a range of unique characteristics which differ from those of research articles and other scientific genres within the framework of written medical discourse. It is to be emphasized that it is highly important for medical professionals to master the major stylistic principles and communicative intentions of medical case report as a genre in order to share their findings with fellow researchers from all over the world. Hence, in the process of training future medical researchers, the analysis of the basic mechanisms of writing a medical case report should be an integral part of the curricula in English for Specific Purposes at universities.

## Background

Within the framework of written medical texts, case reports are traditionally classified into one of the major groups of medical discourse (along with research papers, review articles, and editorials) [1]. As a matter of fact, medical case reports (MCRs) constitute a highly valuable genre of medical literature, since “there is nothing like a good case study for arousing interest, gaining attention, ensuring encouragement, and enabling participation” [2]. Case reports are commonly considered “stepping stones” for further clinical research: “prospective, retrospective and observational randomized controlled trials are always constructed on the basis of data obtained from individual patients” [3]. Apart from their significant role in the dissemination and promotion of medical knowledge, case reports are also valuable in terms of their pedagogic and ethic potential due to their “inextricable connection between narrative and moral knowledge and experience” [2]. It is crucial for medical professionals to take into account all these peculiarities in order to be able to produce effective English language MCRs since they are an indispensable tool for the dissemination of medical knowledge all over the world.

Hence, this genre is exceptionally important as an effective tool of research advancement and for the training of future doctors, and therefore needs careful examination and analysis. The prevalence of English as not only the lingua franca, but as the important transmission medium of scientific knowledge in our time compels professionals in all spheres of science and technology to render their works in English in order to be understood and acknowledged. Thus, the ability to produce the effective English language discourse of MCRs is a vital prerequisite for the dissemination and enhancement of medical knowledge all over the world.

## Methods

Taking the abovementioned into consideration, it is obvious that MCRs need careful examination in terms of their genre and stylistic features which have not yet been thoroughly investigated. The objective of the research is to analyze the genre and stylistic features of MCRs in terms of their lexico-grammatical structure and, in such a way, to provide guidelines for medical professionals for producing effective MCRs. The material of the research is the corpus of MCRs from *Journal of Medical Case Reports*: 15 case reports, published within the last 5 years (2011 to 2016). The selected MCRs will be considered using the method of lexico-grammatical and stylistic analyses, largely focusing on the most significant peculiarities. The use of active and passive voice and the choice of particular verb tenses and pronouns will be subjected to quantitative examination to determine their frequency. In addition, the detected features will be examined with regard to their contextual, structural, and narrative role.

## Results

MCRs are traditionally structured in abstract, introduction, case presentation, discussion, conclusion, informed consent and references. As a rule, the length of a MCR largely depends on the communicative purpose. However, brevity and conciseness are usually the most distinctive features of MCRs as a genre. In general, the author’s aim is to transfer the maximum amount of important information using the minimal linguistic tools. Another unique characteristic of MCRs as compared with other genres of medical discourse is their narrative style. That is to say, the major objective of MCRs is to “narrate”, that is, to describe, an interesting case to fellow researchers in order to warn them or to improve treatment techniques. Consequently, MCRs perform instructive and educational functions, and therefore are aimed at: (1) describing a condition for the first time, and/or (2) warning other physicians. These stylistic features determine the entire structure of MCRs, as well as the choice of lexical units.

It is necessary to admit that the use of simple past tense is a predominant feature of MCRs. This peculiarity results from the very nature of MCRs as narrative (that is, “storytelling”) texts: their primary aim is to give an account of past events. Another important aspect of MCRs is the use of active sentences, contrary to other medical scientific genres, such as research articles. In terms of academic writing, the passive voice and impersonal “it” constructions “depersonalize text and create an impression of the writer’s distance and objectivity” [4], thus providing the necessary effect of academic detachment. However, this is not the case with MCRs. Obviously, this peculiarity is also associated with the narrative style, as well as with the educational intention of MCRs as a genre: the authors of MCRs describe the unusual problem they have faced and handled, thus sharing their experience and a lesson they have learned from it.

Hence, MCRs are notable for their use of active sentences in the simple past tense (309 cases) [3, 5–18]: “We *maintained* anesthesia with inhaled desflurane at 5% and intravenously *administered* remifentanil at 0.2 μg/kg/min in a fraction of inspired oxygen of 0.45 (…) At that moment, we *administered* 20 mg ephedrine, 300 μg phenylephrine, and 0.03 μg/kg/min norepinephrine to maintain adequate blood pressure (…) We *did not detect* any problems with his respiratory parameters (…) His systolic blood pressure *remained* at 40 mmHg for 10 min; we *performed* chest compressions to maintain his blood pressure” [5]; “We *did not find* cases with such an evolution in the literature, but we found some cases of spontaneous expulsion of ileal lipoma per rectum” [6]; “We *found* electrogram amplitude to be normal throughout the right ventricle (…) In this case we *used* cryoablation to avoid the pain associated with radiofrequency delivery” [7]; “An extraoral examination *revealed* a diffuse swelling of her left nasal alar base without tenderness” [8]; “Two patients with EHPVO *presented* with micrognathia, restricted mouth openings, and facial asymmetry” [9]; “Brain magnetic resonance imaging (MRI) *did not identify* any abnormalities, but an ophthalmic examination *revealed* vertical supranuclear gaze palsy (VSGP), and analysis of peripheral blood and bone marrow biopsies *identified* vacuolated histiocytes” [10]; “…we *considered* electroconvulsive therapy; clinicians may opt for electroconvulsive therapy at an earlier stage in similar psychiatric emergencies (…) We *hypothesized* that our patient’s poor clinical response was from a genetic polymorphism in the drug-metabolizing activity of cytochrome P450 enzymes” [11].

However, it is necessary to point out that passive voice constructions can also be met in MCRs. In particular, they are used for the purpose of giving recommendations and warning other physicians (that is, implementation of the instructive function). In this context, the modal verb “should” plays a significant role as well (15 cases in the analyzed MCRs): “The psychological aspect of this condition *should not be underestimated* because he was ashamed of his front teeth and was not able to smile” [12]; “IMA malposition is a rare but potentially lethal complication of CVC that *should be considered* in risk factor assessments and the management of severe complications resulting from CVC” [13]; “…however, the risk of infection *should be weighed* against the advantages of this procedure” [8]; “We hypothesize that a difficult intubation *should be anticipated* in these patients” [9].

The present perfect tense is generally used to render the author’s reflections on the problem in a broader context (for instance, the results of other research, which occur primarily in the “Background” section of MCRs). The research has identified 180 cases of present perfect constructions (103 active and 77 passive sentences): “There *have been* a number of previous reports of CVC malpositioning in the internal mammary vein”; “Numerous complications of central venous catheterization *have been reported*” [13]; “Other studies *have demonstrated* the ability of ablation to prevent recurrence of VF in patients with a structurally normal heart, but these studies also *have been* of small patient cohorts followed only for a few years” [7]; “Few cases of bilateral nasoalveolar cysts have been described” [8].

In addition, the past perfect tense is applied to describe earlier events from the case history (a total of 34 past perfect constructions: 28 active and 6 passive sentences): “Of note, he *had undergone* a THA 31 days prior to his transfer…” [14]; “Four months later, his proteinuria *had reduced* to 1.4 grams/day, creatinine *had improved* to 155 μmol/L” [15]; “A computed tomography (CT) of her abdomen *had shown* nonspecific findings suggestive of colitis” [16]; “Two weeks previously, she *had given* birth at 40 weeks … there had been no antenatal symptoms … our patient *had complained* of dysuria … the baby had not required hospitalization” [17].

At the same time, our research has demonstrated the relatively seldom use of the future tense in MCRs (a total of 16 cases: 13 active and 3 passive sentences). Occasionally, the authors use it in the “Conclusions” section to provide a perspective for further research or a prognosis: “An ultrasound-guided approach rather than the use of a landmark technique to insert CVC *will help*” [13]; “Patients *will tolerate* the procedure with adequate airway preparation using topical anesthesia” [9]; “Until the effectiveness of ablation for the prevention of VF has been confirmed in a large cohort of such patients followed for a substantial duration, we *will continue* to recommend ICD therapy for most survivors of VF arrest even if ablation is also performed” [7]; “The tooth *will preserve* the remaining alveolar ridge and help the adolescent psychologically” [12].

The use of the present simple tense is also comparatively infrequent and it is generally used only in the opening section of MCRs (23 cases in the analyzed discourse): “We report a rare case of a CVC tip malpositioned in the right internal mammary artery (IMA)” [13]; “*We describe* the case of a 30-year-old man from the north of Morocco with no medical or surgical history and no family history of rectal disease” [6]; “*We describe* a case in which RVOT ectopy was complicated by ventricular fibrillation (VF) arrest despite the absence of any of the recognized risk factor for cardiac arrest and despite an apparently positive response to pharmacological therapy” [7]; “Here, *we present* two cases where difficult intubation was anticipated, and in which, despite trying various devices to visualize and secure the airway, only the use of a Macintosh blade proved successful” [9]; “*We report* a case of an acute psychotic relapse in response to polydrug use most notable for multiple recent binges of crystal methamphetamine” [11].

As one can observe from the examples above, the direct manner and personal style of MCRs are vividly embodied in the wide use of the first person plural personal pronoun. The present research has detected 77 cases of using the pronoun “we” in the analyzed MCRs. As a matter of fact, the use of “we” emphasizes the fact of joint authorship: “sole authorship should rarely be undertaken, instead the support and critical appraisal of a number of colleagues, as well as clinical mentors, offers the most likely team to ensure a strong contribution to literature” [19]. Furthermore, the patients are usually referred to as third person pronouns. This narrative strategy is aimed primarily at the protection of patients’ personal information. Hence, all relevant data (for example, a patient’s sex and age) are provided in the first sentence of MCRs, with subsequent referencing as “he” or “she”, so that the patients could not be identified. The research has found 153 cases of referring to patients as “he” or “she” in the analyzed discourse. The following examples demonstrate this strategy:

1. Case presentation: “A 30-year-old man from the north of Morocco presented with rectorrhagia and constipation”. Discussion: “*He* presented with a 1-year history of rectorrhagia and constipation (…) *He* was hospitalized in our surgical department when he defecated spontaneously the tumor mass. (…) At the end of radiotherapy, *he* had follow-up consultation every 3 months (…) After 1 year of surveillance, *he* has not presented any clinical symptoms and pelvic magnetic resonance imaging was normal” [6].
2. Case presentation: “We report the case of a 54-year-old Caucasian woman with symptomatic right ventricular outflow tract arrhythmias without structural heart disease who suffered a ventricular fibrillation arrest without prior malignant clinical features”. Discussion: “At the time of presentation, *she* was taking no medication (…) *She* was discharged on a slow-release preparation of verapamil 240 mg daily for control of symptoms (…) One month later, our patient was readmitted after suffering an out-of-hospital VF cardiac arrest from which *she* was successfully resuscitated (…) On recovery, our patient accepted our recommendation that she undergo ablation of the focus of ventricular ectopy but *she* refused implantable cardioverter defibrillator (ICD) therapy” [7].

Thus, while personal pronouns are for the most part avoided in other scientific genres, they constitute a predominant feature of MCRs.

## Discussion

As one can easily observe, the abovementioned lexico-grammatical features of MCRs differ from research articles and other scientific genres. For instance, unlike the objective reportage of research articles, MCRs provide a personal “storytelling” description. Hence, in the process of training future medical researchers, the study of these stylistic peculiarities and their communicative purposes should be an integral part of the curricula in English for Specific Purposes at universities.

## Conclusions

MCRs as a genre possess a range of unique characteristics, such as: narrative style, conciseness, and brevity which result from the authors’ aspiration to put their messages to colleagues as quickly and precisely as possible; personal tone, stipulated by sharing one’s own experience, as against the detached academic style which is typical of research articles; and educational and instructive intentions, aimed at warning other physicians and improving patient management. These communicative features determine the choice of lexical units and grammatical structures in the analyzed discourse: the dominance of active voice sentences, past simple tense, personal pronouns, and modal verbs. This research has also detected the occasional use of the present perfect, present simple, and future simple tenses and the passive voice which also serve the particular communicative purposes of MCRs. It is our belief that further genre and style analysis of MCRs will help medical professionals to effectively textualize their research results into the MCR genre and thus make their unique contribution to the development of medical science.

## Abbreviations

CT: Computed tomography
CVC: Central venous catheter
EHPVO: Extra-hepatic portal vein obstruction
ICD: Implanted cardioverter defibrillator
IMA: Internal mammary artery
MCR: Medical case report
MRI: Magnetic resonance imaging
RVOT: Right ventricular outflow tract
THA: Total hip arthroplasty
VF: Ventricular fibrillation
VSGP: Vertical supranuclear gaze palsy
